# Establishment of *Culex modestus* in Belgium and a Glance into the Virome of Belgian Mosquito Species

**DOI:** 10.1128/mSphere.01229-20

**Published:** 2021-04-21

**Authors:** Lanjiao Wang, Ana Lucia Rosales Rosas, Lander De Coninck, Chenyan Shi, Johanna Bouckaert, Jelle Matthijnssens, Leen Delang

**Affiliations:** a KU Leuven Department of Microbiology, Immunology and Transplantation, Rega Institute for Medical Research, Laboratory of Virology and Chemotherapy, Leuven, Belgium; b Laboratory of Viral Metagenomics, Rega Institute for Medical Research, KU Leuven, Leuven, Belgium; University of Texas Southwestern Medical Center

**Keywords:** Belgium, *Culex modestus*, haplotype, mosquito, virome

## Abstract

Culex modestus mosquitoes are considered potential transmission vectors of West Nile virus and Usutu virus. Their presence has been reported across several European countries, including one larva detected in Belgium in 2018. In this study, mosquitoes were collected in the city of Leuven and surrounding areas in the summers of 2019 and 2020. Species identification was performed based on morphological features and partial sequences of the mitochondrial cytochrome oxidase subunit I (COI) gene. The 107 mosquitoes collected in 2019 belonged to eight mosquito species, Culex pipiens (24.3%), Cx. modestus (48.6%), Cx. torrentium (0.9%), Culiseta annulata (0.9%), Culiseta morsitans (0.9%), Aedes sticticus (14.0%), Aedes cinereus (9.3%), and Anopheles plumbeus (0.9%), suggesting the presence of an established *Cx. modestus* population in Belgium. The collection of *Cx. modestus* mosquitoes at the same locations in 2020 confirmed their establishment in the region. Haplotype network analysis of the COI sequences for *Cx. modestus* showed that the Belgian population is rather diverse, suggesting that it may have been established in Belgium for some time. The Belgian *Cx. modestus* population was most closely related to populations from the United Kingdom and Germany. Characterization of the virome of the collected mosquitoes resulted in the identification of at least 33 eukaryotic viral species. Nine (nearly) complete genomes belonging to 6 viral species were identified, all of which were closely related to known viruses. In conclusion, here, we report the presence of *Cx. modestus* in the surrounding areas of Leuven, Belgium. As this species is considered to be a vector of several arboviruses, the implementation of vector surveillance programs to monitor this species is recommended.

**IMPORTANCE** *Culex modestus* mosquitoes are considered to be a potential “bridge” vector, being able to transmit pathogens between birds as well as from birds to mammals, including humans. In Belgium, this mosquito species was considered absent until the finding of one larva in 2018 and subsequent evidence of a large population in 2019 to 2020 described here. We collected mosquitoes in the summers of 2019 and 2020 in the city of Leuven and surrounding areas. The mosquito species was identified by morphological and molecular methods, demonstrating the presence of *Cx. modestus* in this region. The ability of mosquitoes to transmit pathogens can depend on several factors, one of them being their natural virus composition. Therefore, we identified the mosquito-specific viruses harbored by Belgian mosquitoes. As *Cx. modestus* is able to transmit viruses such as West Nile virus and Usutu virus, the establishment of this mosquito species may increase the risk of virus transmission in the region. It is thus advisable to implement mosquito surveillance programs to monitor this species.

## INTRODUCTION

The mosquito species Culex modestus was described for the first time by Eugenio Ficalbi in northern Italy in 1889 (1) and is considered a rare species. In Europe, this species is distributed mainly in southern and central European countries. Field collection studies have reported the presence of Cx. modestus in France, Spain, Portugal, Germany, Romania, Serbia, the Czech Republic, and, more recently, in more northern countries such as the United Kingdom, Denmark, and Sweden (2–5). In Belgium, this mosquito species was thought to likely be present given its occurrence in nearby countries (6). Until now, only one larva has been found in 2018 and identified through molecular methods (7). Recent field studies in the United Kingdom have confirmed two characteristics of *Cx. modestus*, (i) its ornithophilic habit, i.e., feeding on resident and migratory bird species (8), and (ii) its mammalophilic and anthropophilic feeding behavior, showing that *Cx. modestus* is also a major human-biting mosquito species similar to Culex pipiens (9). Thus, *Cx. modestus* could play a role in nature as a “bridge” vector, being able to transmit pathogens between birds in an enzootic cycle as well as from birds to mammals, including humans, in an epizootic/epidemic cycle.

Previous studies of different *Cx. modestus* populations in Europe revealed that this species can act as a carrier of different pathogens and is likely able to transmit these pathogens as well. In the south of France, *Cx. modestus* mosquitoes have been found to serve as amplifying vectors for seasonal West Nile virus (WNV), introduced by migratory birds (10). *Cx. modestus* mosquitoes collected in the Danube Delta region (border of Romania and Ukraine) were positive for *Plasmodium* sp. lineage Donana03 (avian malaria) (11). In addition, a prevalence of trypanosomatids of 5.1% was detected in the gut of *Cx. modestus* collected in the Czech Republic between 1998 and 2002 (12). Furthermore, *Cx. modestus* is the vector and reservoir of Lednice virus (LEDV), a rare bunyavirus that causes viremia in wild birds. During the last 60 years, various European countries have reported the presence of LEDV in their *Cx. modestus* mosquito populations (13). Besides LEDV, Tahyna virus (TAHV) has also been isolated from *Cx. modestus* in Czechoslovakia and France (14).

Mosquito surveillance in the United Kingdom started focusing on *Cx. modestus* due to its confirmed establishment and important role in the transmission of WNV and Usutu virus (USUV) (15). The role in WNV transmission in Europe was demonstrated by the detection of WNV in this mosquito species during an outbreak in the Sardinia region of Italy in 2011 (16). During this outbreak, the circulating virus strains belonged to lineage 1. This was the first report of an Italian WNV strain that caused clinical signs in the affected birds. The mosquito survey carried out in this area revealed that these virus strains were found in *Cx. modestus* mosquitoes. During the mosquito seasons of 2015 and 2016, WNV lineage 2 was also detected in *Cx. modestus* mosquitoes collected in the Lednice-Valtice area in southern Moravia (17, 18). Regarding the vector competence of *Cx. modestus* for WNV, this mosquito species was found to be competent to transmit WNV experimentally. More than 90% of *Cx. modestus* mosquitoes developed disseminated infection 14 days after an infectious WNV blood meal (19). Moreover, it is considered an extremely efficient vector given that the dissemination rate and the transmission rate reached 89.2% and 54.5%, respectively, after 14 days of incubation (20).

USUV has also been detected in field-collected *Cx. modestus* mosquitoes, likely cocirculating with WNV (21). USUV is another arbovirus of African origin that is principally transmitted by *Culex* mosquitoes. This virus belongs to the genus *Flavivirus*, along with dengue virus, yellow fever virus, Zika virus, Japanese encephalitis virus, and WNV (22). The virus is maintained in an enzootic cycle between ornithophilic mosquitoes and birds. In Europe, USUV was found in a retrospective analysis of archived tissue samples from bird deaths in the Tuscany region of Italy in 1996 (23). In 2001, USUV-associated death of blackbirds was reported in Austria (24), Germany, and the Netherlands (25, 26). In 2016, numerous wild birds, mainly Eurasian blackbirds (Turdus merula), were affected by a USUV outbreak in Belgium in the provinces of Limburg, Antwerp, and Flemish Brabant (27). In 2017, the virus further spread to the west, and by the summer of 2018, the whole country was affected (28). Despite the recent USUV outbreaks, it is not known which mosquito species are the vectors of USUV in Belgium. To gain insight into which mosquito species might carry clinically relevant viruses, we collected field mosquitoes using BG-Sentinel traps in the city of Leuven and its surrounding areas in three different environment types (urban, periurban, and wetland areas).

To unravel the high diversity of mosquito-specific viruses (MSVs) harbored by Belgian mosquitoes, we performed a metagenomic sequencing approach using the novel enrichment technique of viromes (NetoVIR) protocol (29). The study of viral diversity in mosquitoes is important since MSVs have the potential to modulate the vector competence of mosquitoes for different arboviruses (30). The virome of tropical mosquito species such as Aedes aegypti has been studied extensively. On the other hand, knowledge of the viral diversity in mosquitoes from more temperate regions is still scarce but increasing. For instance, a recent virome study identified novel RNA viruses in Swedish mosquitoes (31). However, the virome of mosquitoes from Western Europe, including Belgium, has not yet been studied. Therefore, we provide a first glance into the virome of mosquitoes collected in Belgium.

## RESULTS

### Mosquito species detected in Leuven, Belgium.

**FIG 1:**
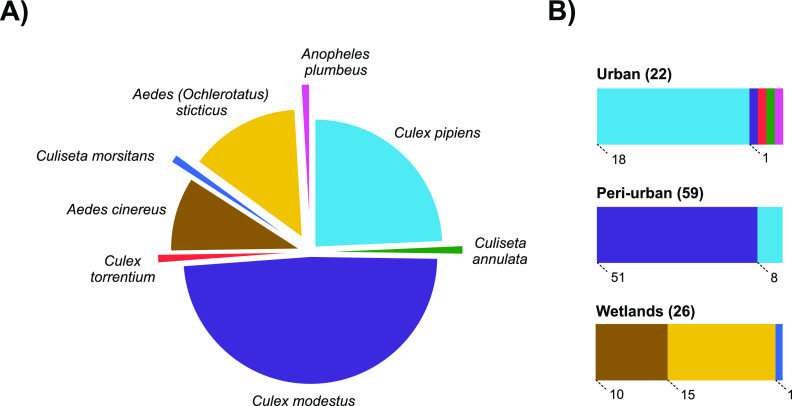
Mosquito species collected in Leuven, Belgium, in 2019. (A) Distribution of mosquito species captured during the summer of 2019 across all locations sampled in Leuven. (B) Distribution of mosquito species across habitat types in Leuven. Mosquito species are marked in different colors. The number of specimens is indicated in the bar chart.

A total of 107 mosquito specimens were collected in three distinct locations in Leuven in the summer of 2019. According to the DNA barcodes generated and morphological features, these mosquitoes belonged to eight mosquito species: Culex pipiens (24.3%), *Cx. modestus* (48.6%), Cx. torrentium (0.9%), Culiseta annulata (0.9%), Culiseta morsitans (0.9%), Aedes sticticus (14.0%), Aedes cinereus (9.3%), and Anopheles plumbeus (0.9%) (Fig. 1A). Surprisingly, *Cx. modestus* accounted for ∼50% of all collected mosquitoes in three different breeding sites. *Culex* species were predominant in urban and periurban areas, whereas specimens found in the water reservoir wetlands belonged mostly to the genus *Aedes* (Fig. 1B).

### Establishment of *Cx. modestus* in Leuven, Belgium.

**FIG 2:**
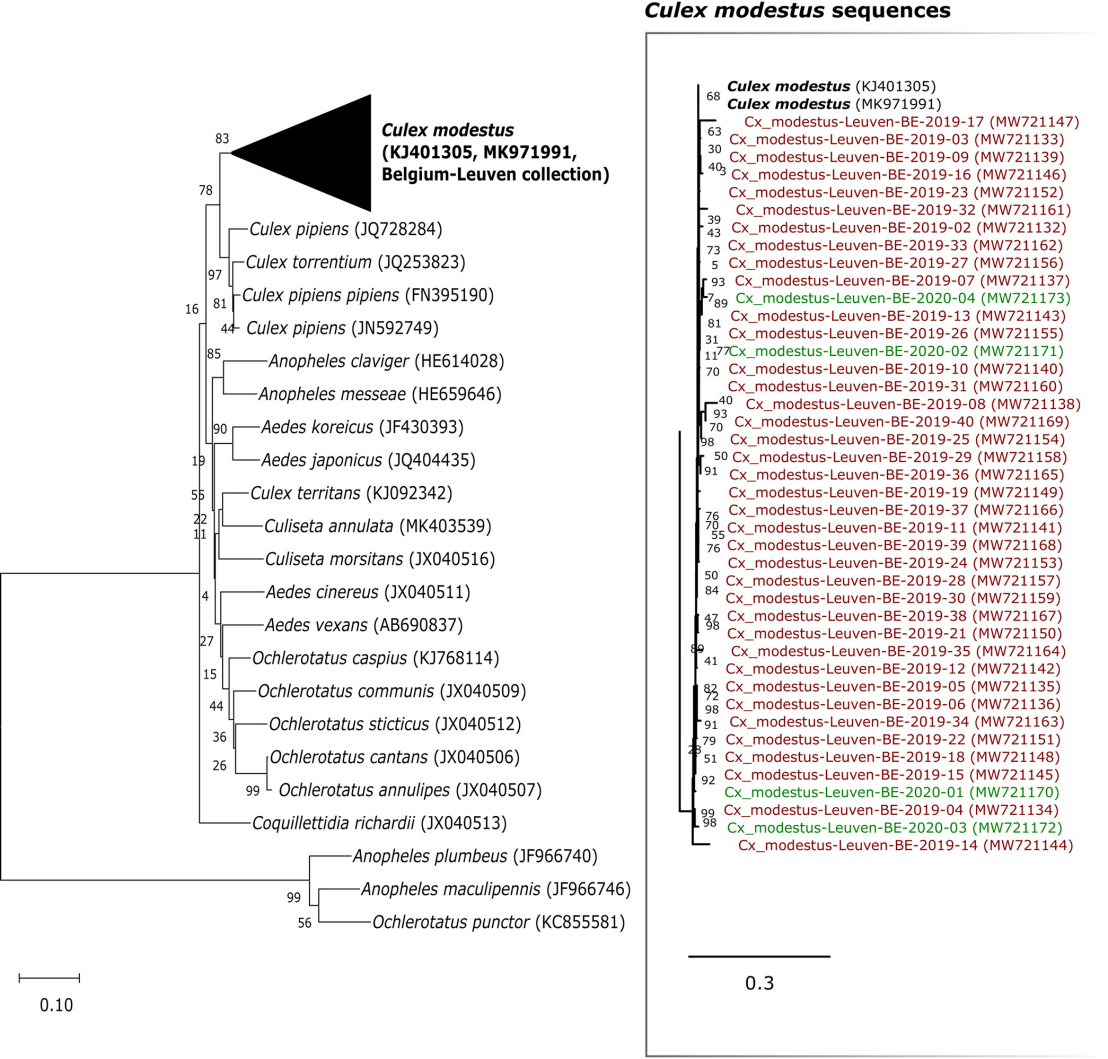
ML tree of the COI sequences of 21 culicid species. Sequences derived from mosquitoes collected in Leuven are collapsed with the reference sequences for *Cx. modestus*. The collapsed branch is expanded in the panel on the right. GenBank accession numbers are in parentheses.

A maximum likelihood (ML) tree was built from the *Cx. modestus* cytochrome oxidase subunit I (COI) barcodes obtained in Leuven and COI sequences of 20 other culicid species described previously (6). *Cx. modestus* barcodes from Leuven clustered with two reference *Cx. modestus* sequences that were included (GenBank accession numbers KJ401305 and MK971991). All sequences for *Cx. modestus* fell within one large well-supported monophyletic cluster, separated from other mosquito species, which suggests that they belong to the same species (Fig. 2). To find out whether *Cx. modestus* is established in the region, field collections were performed in the summer of the consecutive year (2020) using the same geographic locations as the ones used previously. Again, *Cx. modestus* mosquitoes were retrieved (Fig. 2), confirming the establishment of this mosquito species in the area of Leuven.

### Haplotype network of *Cx. modestus* mosquitoes.

The data set analyzed for haplotype inference was constructed by employing 184 *Cx. modestus* partial COI sequences retrieved from the NCBI database corresponding to eight European countries and including 40 partial high-quality COI sequences obtained from the molecular identification of field-collected mosquitoes in Leuven (see Tables S2 to S4 in the supplemental material). Four partial COI sequences from mosquitoes collected during the summer of 2020 were included as well.

10.1128/mSphere.01229-20.2 TABLE S1 *Culex modestus* sequences downloaded from GenBank and employed for the construction of the haplotype network. Download Table S1, PDF file, 0.01 MB. Copyright © 2021 Wang et al. 2021 Wang et al. https://creativecommons.org/licenses/by/4.0/ This content is distributed under the terms of the Creative Commons Attribution 4.0 International license.

10.1128/mSphere.01229-20.3 TABLE S2 Haplotype frequencies among populations of *Culex modestus* from 9 countries in Europe. Download Table S2, PDF file, 0.09 MB. Copyright © 2021 Wang et al. 2021 Wang et al. https://creativecommons.org/licenses/by/4.0/ This content is distributed under the terms of the Creative Commons Attribution 4.0 International license.

Among the 228 COI sequences (639 bp), 97 haplotypes were found. The majority of haplotypes (88) were present only in the country of origin, while only 9 haplotypes were shared by two or more countries. Haplotype diversity ranged from 0.8182 in Spain to 1.000 in Denmark, Portugal, Serbia, and Sweden (Table 1). This analysis revealed that haplotype diversity in Belgium was the second highest (0.9852) of all countries screened, followed by the United Kingdom (0.9252) and Germany (0.9013). Nucleotide diversity estimations ranged from 0.0058 in Spain to 0.0270 in Belgium. Belgium exhibited a nucleotide diversity of 0.0270, which can be considered moderate but which is the highest in all included European countries.

**TABLE 1:** Haplotype and nucleotide diversity of *Cx. modestus* from 9 countries in Europe

Location of population	No. of samples	No. of haplotypes	Mean haplotype diversity ± SD	Mean nucleotide diversity ± SD
Belgium	44	33	0.9852 ± 0.0082	0.0270 ± 0.0136
Denmark	7	7	1.0000 ± 0.0764	0.0174 ± 0.0103
France	28	11	0.8598 ± 0.0462	0.0069 ± 0.0039
Germany	42	17	0.9013 ± 0.0278	0.0113 ± 0.0060
Portugal	2	2	1.0000 ± 0.5000	0.0065 ± 0.0073
Serbia	4	4	1.0000 ± 0.1768	0.0182 ± 0.0125
Spain	22	8	0.8182 ± 0.0586	0.0058 ± 0.0034
Sweden	5	5	1.0000 ± 0.1265	0.0085 ± 0.0058
UK	74	28	0.9252 ± 0.0176	0.0107 ± 0.0057

### Mitochondrial DNA genealogy of *Cx. modestus*.

**FIG 3:**
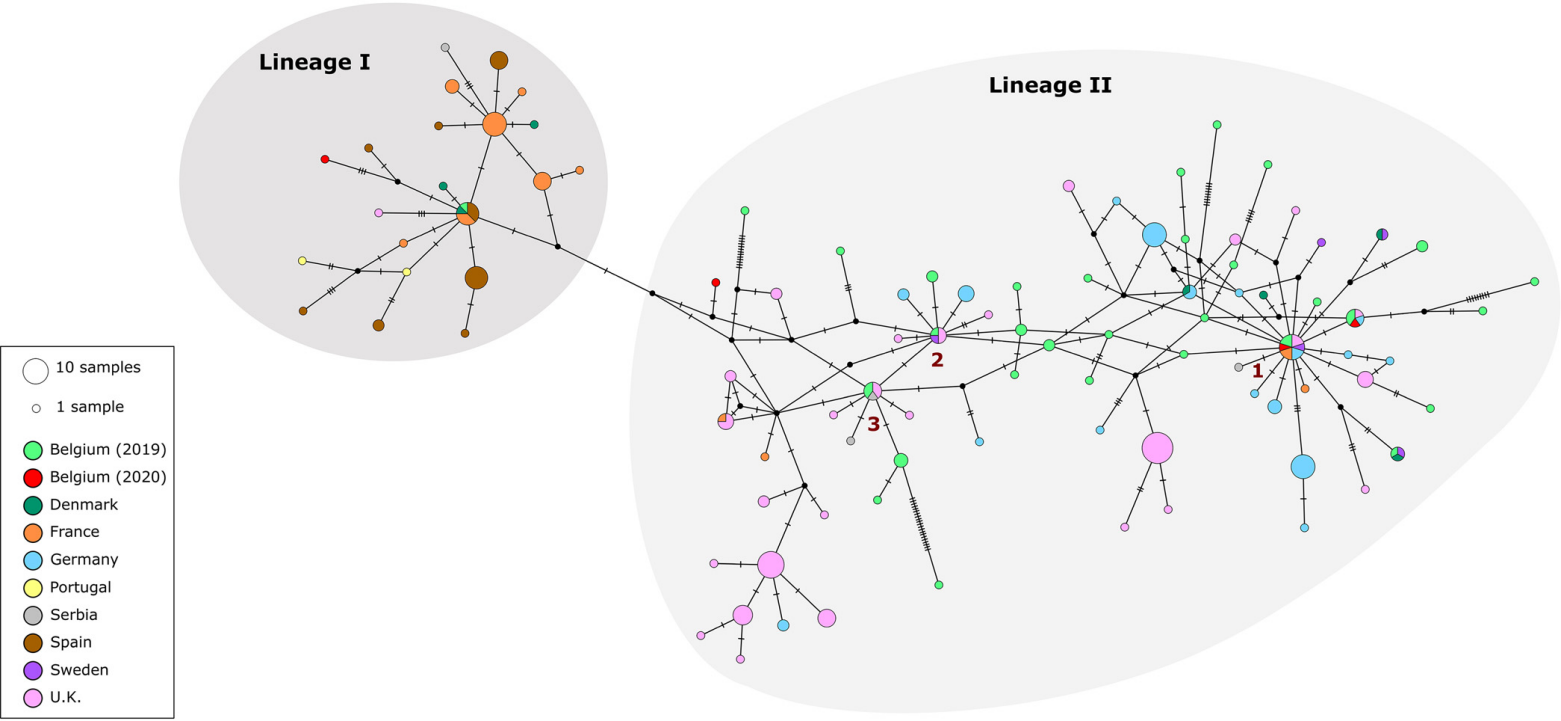
Median-joining network constructed with 228 COI sequences of *Cx. modestus* from 9 countries in Europe. Each circle represents a haplotype. The size of the circle corresponds to the number of specimens sharing that specific haplotype. Each country is represented by a color, described in the key. Mosquito collections in Belgium are separated per year to visualize the allocation of haplotypes in the network. The gray backgrounds represent both lineages found and the distinction of these two groups.

The median-joining (MJ) network displayed the ancestry of *Cx. modestus* mosquitoes (Fig. 3), where two lineages were visualized, separated by 1 mutation step. Haplotypes from Spain and Portugal were found uniquely in lineage I, while haplotypes from Germany, the United Kingdom, Belgium, and Sweden predominated in lineage II. Haplotypes from France, Serbia, and Denmark were scattered across both lineages. The majority of haplotypes that were found in Belgium were located between three central haplotypes of lineage II, which contain samples from several countries: one is shared by Belgium, the United Kingdom, and Serbia (Fig. 3, 3); another one is shared by Belgium, the United Kingdom, and Sweden (Fig. 3, 2); and the largest one is shared by Belgium, Germany, the United Kingdom, France, and Sweden (Fig. 3, 1). Haplotypes found in mosquitoes collected in Leuven during the summer of 2020 were observed in both lineage I (1 haplotype) and lineage II (3 haplotypes).

### A peek into the virome of Belgian mosquitoes.

**FIG 4:**
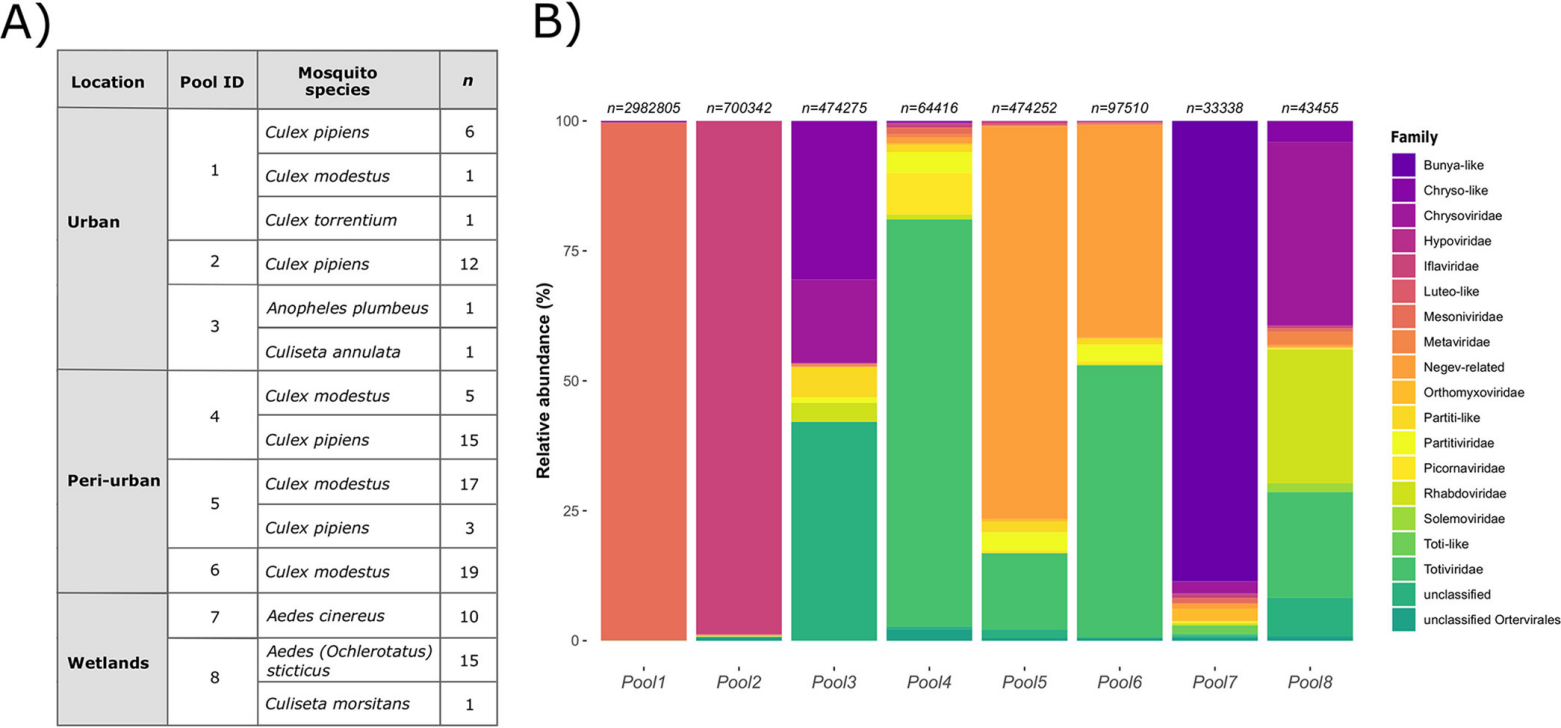
Summary information and viral composition of sequenced samples. (A) Location, mosquito species, and number of specimens present in each of the sequenced pools. (B) Bar plots representing the abundance of reads belonging to distinct viral families per pool. The number of eukaryotic viral reads per pool is given on top of each bar.

We characterized the virome of 107 mosquitoes’ abdomens, divided into eight pools according to their morphological identification and representing the three different habitat types mentioned above (Fig. 4A). A total of 44,002,358 reads were obtained from all mosquito pools. Most reads (21,602,296; 49.1%) belonged to the urban group. Mosquitoes collected in periurban and wetland areas generated 13,891,285 (31.6%) and 8,508,777 (19.3%) reads, respectively.

In all pools, the proportion of reads mapping to the order Diptera ranged from 40.8 to 77.7%. Regarding the bacterial reads, the wetland samples had a higher mean proportion (3.83%), followed by the urban samples, with 2.03%, while the periurban samples presented <1% of reads mapping to bacteria. The viral component was more variable, with an observable ascending trend when moving from the wetlands to periurban and urban areas. Wetland samples gathered a low proportion of viral reads (<2%), whereas viral reads in periurban areas accounted for 1.28 to 7.19%. Finally, reads mapping to the viral component comprised 7.45 to 44.69% of the urban samples.

After filtering the viral reads for eukaryotic viral species, the relative abundances in the mosquito pools are shown in Fig. 4 per viral family. The *Culex* pools in the urban area were completely dominated by one viral family (*Mesoniviridae* and *Iflaviridae* for pool 1 and pool 2, respectively). The periurban samples contained mostly viral reads from a Negev-related virus, namely, Yongsan negev-like virus 1, and from the *Totiviridae* family, with Culex inatomii totivirus being the most abundant viral species. In the wetland Ae. cinereus pool, on the other hand, an unclassified bunya-like virus was the most abundant.

### Comparing the eukaryotic viromes across habitat types and mosquito genera.

**FIG 5:**
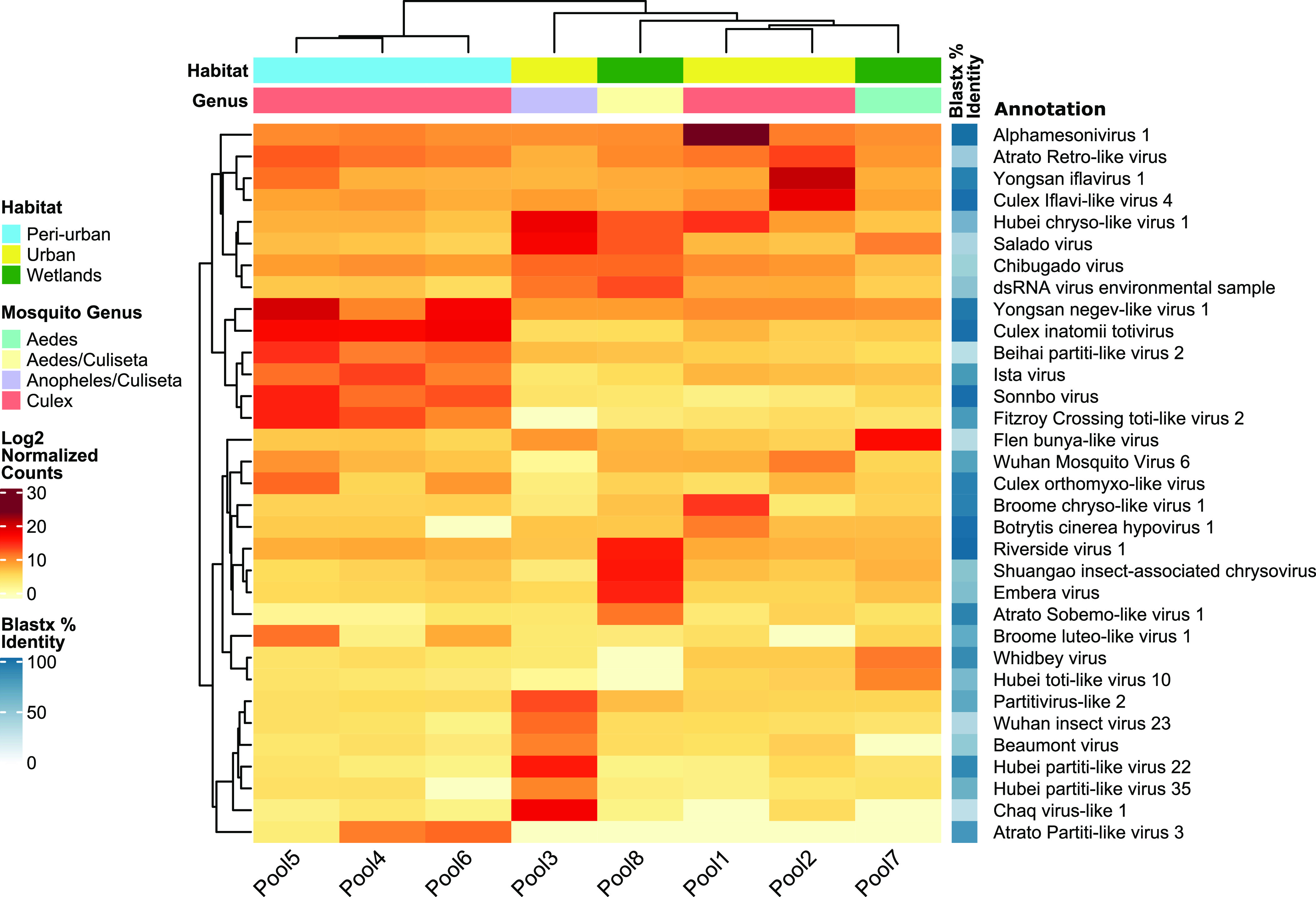
Heat map of normalized read counts for eukaryotic viruses. The heat map shows the normalized count on a log_2_ scale of reads mapping to the assembled contigs of each eukaryotic virus. Next to the taxonomic annotation, obtained by DIAMOND and KronaTools, the average BLASTx identity for all contigs representing a viral species is depicted by the shaded blue boxes. Hierarchical clustering of the columns is based on the Bray-Curtis distance matrix calculated from the normalized read counts.

To compare the eukaryotic viromes of our samples, we mapped all trimmed and decontaminated reads back to the selected viral contigs, extracted the abundance table, and subsequently constructed a heat map with the normalized counts for each viral species on a log_2_ scale (Fig. 5). In total, 33 eukaryotic viral species could be detected across all samples (a viral species was considered present if it had at least one contig of >1,000 bp and if more than 500 reads mapped to it). According to the Bray-Curtis distance matrix, the eukaryotic viromes of the *Culex* mosquito pools clearly clustered together per habitat type. However, except for the periurban *Culex* pools, each remaining pool had a more unique viral composition, and only a small number of viruses were significantly shared between samples. Nevertheless, the periurban mosquito pools had a majority of viruses in common, such as Culex inatomii totivirus and Yongsan negev-like virus 1, which were shared with high abundances, while Ista virus, Sonnbo virus, and Fitzroy Crossing toti-like virus 2 were common in lower abundances.

### Recovery of (nearly) complete meta-assembled genomes.

**FIG 6:**
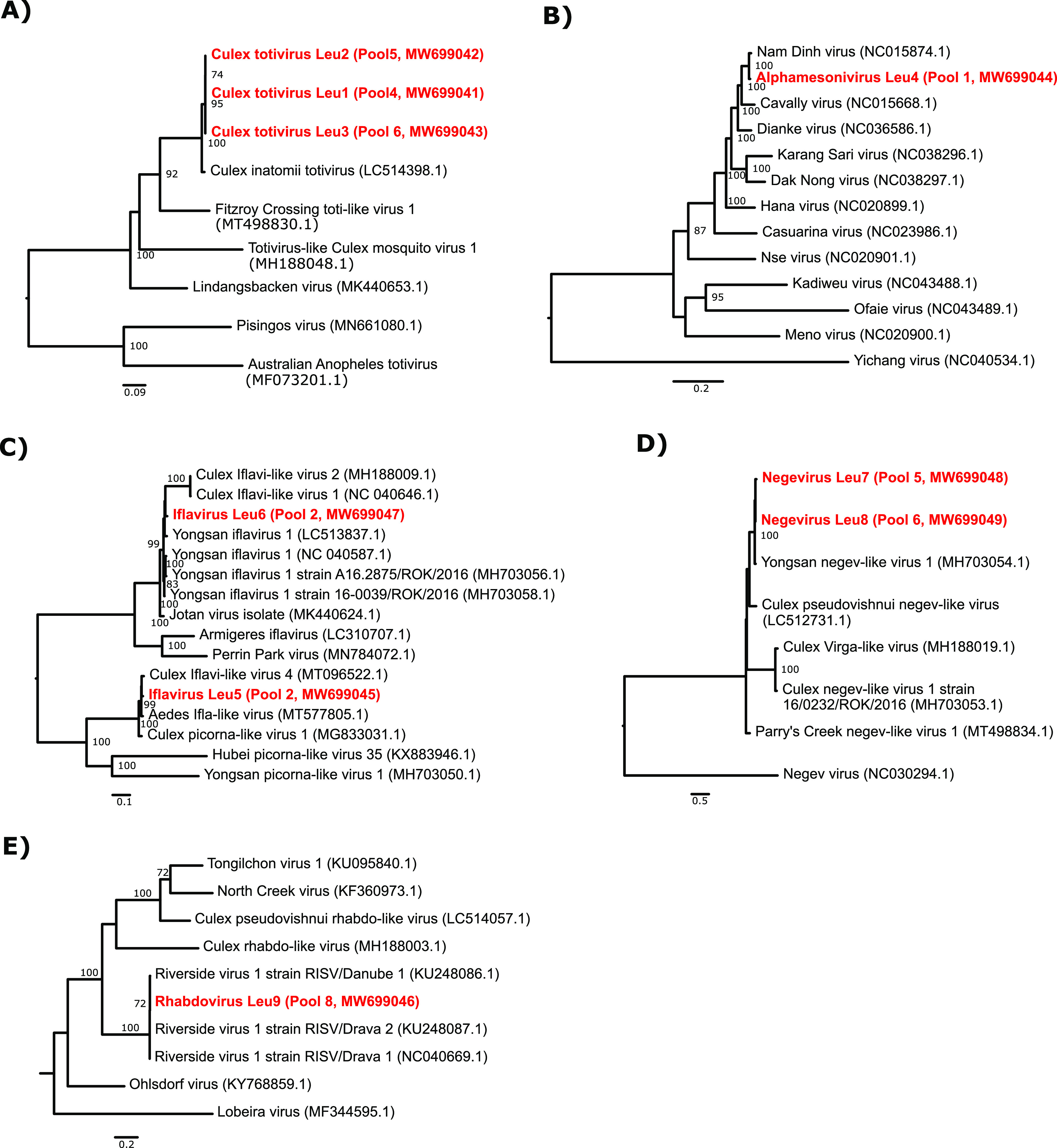
(Nearly) complete meta-assembled genomes identified in mosquitoes collected during the summer of 2019. Bootstrap support values are shown next to the nodes. Complete MAGs are colored in red. (A) Midpoint-rooted ML tree of all complete genomes related to Culex inatomii totivirus, selected after BLASTn analysis. (B) Midpoint-rooted ML tree of all *Mesoniviridae* family members. (C) Midpoint-rooted ML tree of all complete genomes related to our Yongsan iflavirus 1 and Culex iflavi-like virus 4 genomes, selected after BLASTn analysis. (D) ML tree of all complete genomes related to Yongsan negev-like virus 1, selected after BLASTn analysis. Negevirus was used as the outgroup. (E) Midpoint-rooted ML tree of all complete genomes related to the recovered Riverside virus 1. GenBank accession numbers are in parentheses.

In total, we managed to recover 9 (nearly) complete genomes of 6 viral species in our metagenomic data. These viral species belong to the following families: *Totiviridae* (Culex inatomii totivirus in pools 4, 5, and 6), *Iflaviridae* (Yongsan iflavirus 1 and Culex iflavi-like virus 4 in pool 2), *Mesoniviridae* (Alphamesonivirus 1 in pool 1), *Rhabdoviridae* (Riverside virus 1 in pool 8), and unclassified Negev-related viruses (Yongsan negev-like virus 1 in pools 5 and 6), and their phylogenetic relatedness to closely related reference strains is shown in Fig. 6 (Table S5).

10.1128/mSphere.01229-20.4 TABLE S3 Haplotypes among populations of *Culex modestus* from 9 countries in Europe. Download Table S3, PDF file, 0.01 MB. Copyright © 2021 Wang et al. 2021 Wang et al. https://creativecommons.org/licenses/by/4.0/ This content is distributed under the terms of the Creative Commons Attribution 4.0 International license.

10.1128/mSphere.01229-20.5 TABLE S4 Summary information of the complete genomes identified in this study. Download Table S4, PDF file, 0.01 MB. Copyright © 2021 Wang et al. 2021 Wang et al. https://creativecommons.org/licenses/by/4.0/ This content is distributed under the terms of the Creative Commons Attribution 4.0 International license.

10.1128/mSphere.01229-20.6 TABLE S5 BLASTx percent identities of annotated *de novo*-assembled contigs. Download Table S5, PDF file, 0.03 MB. Copyright © 2021 Wang et al. 2021 Wang et al. https://creativecommons.org/licenses/by/4.0/ This content is distributed under the terms of the Creative Commons Attribution 4.0 International license.

### dsRNA viruses: *Totiviridae.*

The *Totiviridae* family of double-stranded RNA (dsRNA) viruses is known to infect fungi, plants, and invertebrates. In this study, we found Culex totivirus Leu1 (GenBank accession number MW699041), Leu2 (accession number MW699042), and Leu3 (accession number MW699043) (98.3% average BLASTx identity with Culex inatomii totivirus [GenBank accession number LC514398.1]) in all periurban mosquito pools. This novel totivirus was recently described in Cx. inatomii mosquitoes in Japan (32), and our findings now confirm its association with mosquitoes as a host.

### Positive-sense single-stranded RNA [(+)ssRNA] viruses. (i) *Mesoniviridae*.

When constructing a phylogenetic tree of the metagenomic assembled genomes (MAG) annotated as Alphamesonivirus 1 (99.7% BLASTx identity [GenBank accession number MH520101.1]), together with all reference sequences of the *Mesoniviridae* family, our complete Alphamesonivirus Leu4 genome (GenBank accession number MW699044) formed a clade with Nam Dinh virus and Cavally virus. Both alphamesoni 1 viruses are frequently linked to mosquitoes (33, 34). Interestingly, all known members of the *Mesoniviridae* family infect mosquito hosts.

### (ii) *Iflaviridae*.

Iflaviruses are a well-known group of picorna-like viruses that exclusively infect arthropods (35). We found two complete genomes of iflaviruses (iflavirus Leu5 [GenBank accession number MW699045] and iflavirus Leu6 [accession number MW699047], having 98.3 and 97.1% BLASTx identities with Culex iflavi-like virus 4 [accession number MT096522.1] and Yongsan iflavirus 1 [accession number NC_040587.1], respectively) in an urban mosquito pool consisting entirely of *Cx. pipiens* mosquitoes.

### (iii) Negev-related viruses.

Negevirus is a proposed taxon for diverse and geographically widely distributed insect-specific viruses isolated from mosquitoes and phlebotomine sandflies (36). We recovered 2 full genomes annotated as Yongsan negev-like virus 1 (average of 95.1% BLASTx identity [GenBank accession number MH703054.1]) from two periurban mosquito pools that mainly contained *Cx. modestus* mosquitoes, named Negevirus Leu7 (GenBank accession number MW699048) and Leu8 (accession number MW699049).

### (−)ssRNA viruses: *Rhabdoviridae*.

Rhabdoviruses are a diverse group of negative-sense ssRNA [(−)ssRNA] viruses known to infect both vertebrates and invertebrates as well as plants (37). Riverside virus 1 was first described in *Ochlerotatus* sp. mosquitoes in central Europe (38), and in this study, it was also detected (98.2% BLASTx identity [GenBank accession number KU248086.1]). Rhabdovirus Leu9 (GenBank accession number MW699046) was identified in a pool containing mostly *Ochlerotatus* mosquitoes. This suggests a restricted host species range as, to date and to our knowledge, this virus has not yet been found in other mosquito species or other hosts.

## DISCUSSION

A national mosquito inventory between 2007 and 2010 (MODIRISK project) showed that the mosquito fauna in Belgium is composed of 23 mosquito species belonging to five traditionally recognized genera, including 21 indigenous and 2 exotic species (Ae. koreicus and Ae. japonicus) (39). The five most abundant species were *Cx. pipiens* (61.62%), Coquillettidia richiardii (15.43%), *Ae. cinereus* (5.04%), Anopheles claviger (3.52%), and Ae. vexans (2.93%) (39). In this mosquito surveillance study performed in Leuven using BG-Sentinel traps, eight mosquito species were collected, of which seven species (*Cx. pipiens*, *Cx. torrentium*, *Culiseta annulata*, *Culiseta morsitans*, Ae. sticticus, *Ae. cinereus*, and *Anopheles plumbeus*) have been reported previously as autochthonous species of Belgium (according to the latest mosquito species checklist [6]). As only one surveillance methodology was used here, this study might undersample the mosquito diversity in Leuven. For future surveillance campaigns, additional types of mosquito traps, besides the BG-Sentinel traps (such as CDC light traps and gravid traps), will be used.

Reports on the detection of *Cx. modestus* in Belgium have been absent until very recently (7). Only one larva has been detected during the latest exotic mosquito survey carried out from 2017 to 2019 (7, 40). In contrast, during our survey in 2019, *Cx. modestus* accounted for almost half of the mosquitoes that were collected, in three different breeding sites. In addition, *Cx. modestus* mosquitoes were reconfirmed at the same collection sites in the summer of 2020. This finding suggests the establishment of this mosquito species in Belgium, potentially introduced from the United Kingdom or Germany. The appearance and spread of *Cx. modestus* in the United Kingdom have been reported only recently as well, although this species seems to be abundantly present in certain regions based on recent surveys (2017 and 2019) (3). The hypothesis for not noticing its presence in the United Kingdom previously probably relies on the misidentification of *Cx. modestus* as other mosquito species such as *Cx. torrentium* (3).

Along with the introduction of a new mosquito species in a region, its potential role in the transmission of arboviruses that could cause disease in animals and humans must be evaluated. The presence of *Cx. modestus* in Belgium could be problematic as it is one of the most important vectors for *Dirofilaria* spp. such as Dirofilaria immitis (41). Furthermore, the coexistence of *Cx. pipiens* and *Cx. modestus*, two important vectors, may increase the risk of transmission of WNV and USUV given the right circumstances. These two viruses are likely to cocirculate in the same habitat, where birds and *Cx. modestus* mosquitoes play their roles as hosts and vectors, respectively (21). In September 2020, the enzootic transmission of WNV in the Netherlands, a neighboring country of Belgium, was confirmed for the first time by detecting simultaneously the presence of the virus in a local common whitethroat, in field-collected mosquito pools, and in humans (42). Given the establishment of *Cx. modestus* in Belgium, it would be advisable to implement vector surveillance for this species. In Europe, the higher biting activity displayed by *Cx. modestus* lasts from July until the beginning of October. However, given the detection of Tahyna virus (an arbovirus) in hibernating *Cx. modestus* mosquitoes in France (14), winter collection can also be considered for the surveillance of mosquito-transmitted pathogens.

In order to examine the genetic structure of the *Cx. modestus* population found in Leuven, we gathered mitochondrial sequences of *Cx. modestus* mosquitoes collected in other countries across Europe and constructed a haplotype network using the MJ method based on 228 partial COI sequences. As recently reported (3), *Cx. modestus* populations across Europe are separated into two lineages. According to this network, most Belgian haplotypes were connected to haplotypes from the United Kingdom and Germany, suggesting that the mosquito population in Leuven, Belgium, could be derived from these two populations. There were three central haplotypes in lineage II that were shared by several countries. In lineage I, there is one central haplotype that was shared by individuals from Denmark, Spain, and Belgium. These data might indicate that *Cx. modestus* mosquitoes belonging to both lineages are present in Belgium, suggesting the occurrence of at least two independent introduction events.

Vector competence of the mosquito can be influenced by several factors. Bacterial symbionts such as *Wolbachia* have the ability to hinder infection by a variety of pathogens such as chikungunya virus, dengue virus, Zika virus, WNV, and malaria-causing *Plasmodium* species in different mosquito species (43). It is possible that viral symbionts discovered in mosquitoes may have a similar effect. For instance, the insect-specific virus Nhumirim virus was shown to inhibit the replication of WNV, St. Louis encephalitis virus, and Japanese encephalitis virus in C6/36 cells (44). As a first step to unveil the role of viral symbionts in the mosquito’s vector competence, we investigated the virome of the collected mosquitoes. Of note, no USUV or WNV was detected in the collected *Culex* mosquitoes. Furthermore, no Lednice virus was detected in the *Cx. modestus* samples, although this mosquito species was reported to be an important Lednice *Orthobunyavirus* vector (13). In total, 33 eukaryotic viral species could be detected across all our samples in this study, and we recovered 9 (nearly) complete genomes of 6 viral species.

When comparing viral hits across the mosquito species and habitat types where they were collected, some similarities could be observed. Mosquito pools belonging to the same genus seemed to have more viruses in common, as shown by the clustering of the *Culex* mosquito pools or the distinct virome profile presented by the pool composed of *Anopheles*/*Culiseta* (pool 3) compared to the other pools. Additionally, we observed clustering of pools per habitat type. In this case, periurban mosquito pools harbored several viruses in common and in great abundances, such as Culex totivirus Leu1, Leu2, and Leu3 and Negevirus Leu7 and Leu8, closely related to Culex inatomii totivirus and Yongsan negev-like virus 1, respectively. Also, the 6 viral species for which a (nearly) complete genome was recovered were previously reported as, or clustered with, viruses associated with mosquitoes, which might hint at the preservation of a core mosquito virome. However, a larger sampling size is needed to suggest that the virome composition and its abundance differ according to genus, local acquisition and ecosystem, and habitat composition.

When comparing our results with those of a virome study on Cx. quinquefasciatus and Ae. aegypti mosquitoes collected from Guadeloupe, which is the largest island of the French West Indies in the Caribbean, there were two virus species (Hubei toti-like virus 10 and Hubei partiti-like virus 22) found to be shared with Belgian mosquitoes (45). The fact that the same virus species was found in mosquitoes collected in Belgium and Guadeloupe could indicate a widespread global movement and/or long host-virus coevolution. Moreover, several viruses were shared with Northern European Swedish mosquitoes (Whidbey virus, Hubei partiti-like virus 22, Chaq virus-like 1, Ista virus, Wuhan mosquito virus 6, and Sonnbo virus) (31, 46). At the virus family/order level, the relative virome abundance of Swedish *Cx. pipiens* was dominated by the luteoviruses, orthomyxoviruses, and Nam Dinh virus. In contrast, the virome of Belgian *Cx. pipiens* was dominated by *Iflaviridae* (pool 2).

When mosquito samples are pooled, as we did in our study, the virome profile could be strongly skewed by one or a few high-titer virus infections from a single mosquito in the pool. In a study of Swedish mosquitoes, Pettersson et al. (46) reported that 30% of all reads of one of the libraries composed of *Cx. torrentium* mosquitoes were annotated as Nam Dinh virus. From pool 1, we recovered the (nearly) complete genome of Alphamesonivirus Leu4, which is a member of the *Mesoniviridae* family that contains Nam Dinh virus. Considering what was reported in Swedish mosquitoes and that pool 1 was the only pool containing one individual of *Cx. torrentium*, we suggest that Alphamesonivirus Leu4 might have been harbored by this mosquito species as it was not found in any other mosquito pool. In our study, the occurrence of more than one mosquito genus in the same pool was unintentional and resulted from pooling based on morphological identification. For further research, the use of individual mosquito bodies is recommended to perform virome characterization. The feasibility of this approach using single mosquitoes has been evaluated, and no significant differences in total read numbers and viral read proportions were found compared to pooled mosquito samples (45). In addition, processing of individual mosquitoes will more truly indicate prevalence and might provide insights into potential genotype variation between different collection sites.

In conclusion, here, we report the establishment of *Cx. modestus* in the surrounding areas of the city of Leuven, Belgium. The virome of the collected mosquitoes was revealed by a metagenomics approach. As *Cx. modestus* is considered to be a potential vector of WNV, USUV, and other pathogens, surveillance for this mosquito species is recommended.

## MATERIALS AND METHODS

### Ethics statement.

Permits for periurban and wetland mosquito field collections were obtained from the leaders from the Space and Real State division and the Technical Services Department at the University of Leuven (KU Leuven). Permits for field collections in urban habitats were obtained from the landowners.

### Mosquito collections.

Adult mosquitoes were trapped with BG-Sentinel traps (BioGents GmbH, Germany), which were baited with BG-lure (BioGents GmbH, Germany) and contained around 2 kg of dry ice in the isolated box for CO_2_ production. Two traps were rotated in three different habitat types (urban [50°52′41″N, 4°41′21″E], periurban [50°51′N, 4°41′E], and water reservoir wetlands [50°51′N, 4°40′E]) in Leuven and the surrounding areas (see Fig. S1 in the supplemental material).

10.1128/mSphere.01229-20.1 FIG S1 Overview of the collection sites and points representing three distinct habitat types in Leuven: botanic garden (urban), Arenberg Castle (periurban), and wetlands (source, Google Maps). A BG-Sentinel trap at the collection site is displayed in the bottom right corner. Download FIG S1, PDF file, 0.2 MB. Copyright © 2021 Wang et al. 2021 Wang et al. https://creativecommons.org/licenses/by/4.0/ This content is distributed under the terms of the Creative Commons Attribution 4.0 International license.

The parameters to determine each trap location in these habitats were similar to those described previously by Mayi et al. (47). We followed these criteria and the advice of Raf Aerts and his team at the Division of Ecology, Evolution, and Biodiversity Conservation, KU Leuven, on the selection of mosquito collection sites representing different habitat types.

Collections were performed from August to the beginning of October 2019, when the weather was good, avoiding strong wind or heavy rain. Every 24 h, the traps were emptied and repositioned between sunrise and sunset of the next day. Mosquitoes were individually stored at −80°C until species identification. A second collection was performed in August of 2020 in the same geographic locations as the ones described above to confirm the presence of certain species.

### Species identification, sample preparation, and DNA sequencing.

All collected mosquitoes were identified using morphological characteristics (48). Individual thoraces were removed using forceps for molecular identification and homogenized in 100 μl of phosphate-buffered saline (PBS) using tubes with 2.8-mm ceramic beads with a Precellys Evolution homogenizer. Sample preparation was performed by lysing the homogenate at 100°C for 10 min (49). Tissue debris was removed by centrifugation at 12,000 rpm for 3 min, and 50 μl of the supernatant was collected into a new tube. A 710-bp region of the cytochrome oxidase subunit I (COI) mitochondrial gene was the target for amplification by PCR using previously reported primers (50). The presence of the PCR product was checked on a 2% agarose gel by gel electrophoresis. DNA was purified with the Wizard SV gel and PCR clean system (Promega). The DNA concentration of the amplicon was measured by using a NanoDrop instrument (Thermo Fisher), after which samples were sent to Macrogen Europe for Sanger sequencing.

### Mosquito sequence analysis and phylogeny.

Sequences were edited and assembled with BioEdit version 7.2.5 (51) to obtain a single consensus sequence per mosquito. Through the BLAST tool, the generated COI sequences were compared to the NCBI database. Reference COI sequences for all mosquito species considered were selected according to methods described previously by Versteirt and colleagues (52), which employed reference sequences that were registered in the Barcode of Life Data (BOLD) systems, and downloaded from GenBank. For phylogenetic analysis, the COI sequences generated in the study and the reference sequences were aligned with MAFFT v7.471 (53) using the G-INS-I option. The resulting alignment was trimmed by using trimAl v1.4.rev15 (54) with the gappyout setting, and phylogenetic informative regions of the alignment were selected with BMGE v1.12 (55) for phylogenetic inference. Maximum likelihood (ML) trees were constructed using IQ-TREE v2.0.3 (56) with automatic selection of the best nucleotide substitution model and 1,000 ultrafast bootstrap replicates. Finally, trees were visualized using FigTree v1.4.4.

### Haplotype network.

Haplotype inference and nucleotide diversity were calculated in ARLEQUIN version 3.5.2.2 (57). The population genetic data were analyzed using the median-joining (MJ) network algorithm in PopART version 1.7 (58, 59). The COI sequences for *Cx. modestus* included in the haplotype network were retrieved from the NCBI database. These sequences were selected based on the specimen’s country of origin and the length of the COI fragment (50).

### Pool design, sample preparation, and sequencing for virome analysis.

The samples of mosquito abdomens were grouped in pools for sequencing according to the morphological identification of mosquito species by key points and sample location (urban, periurban, and wetlands). Abdomens were homogenized in 600 μl of PBS with 2.8-mm ceramic beads with the Minilys tissue homogenizer, including a negative control (blank tube with PBS).

All pool samples were prepared using the novel enrichment technique of viromes (NetoVIR) sample preparation protocol optimized for viral metagenomics (60, 61). In brief, after homogenization, samples went through a centrifugation-and-filtration step (by using a 0.8-μm filter; Sartorius) to remove pro- and eukaryotic organisms and large organic debris. Next, nuclease treatment (employing Benzonase and micrococcal nuclease) was applied to remove free-floating nucleic acids. Nucleic acids were extracted with the QIAamp viral RNA minikit (Qiagen) to be further randomly amplified using a modified whole-transcriptome amplification 2 (WTA2) kit procedure (Sigma-Aldrich). The products were purified, and libraries were prepared using the NexteraXT library preparation kit (Illumina). Sequencing of the samples was carried out on a NextSeq 500 high-throughput platform (Illumina) for 300 cycles.

### Bioinformatic analysis and identification of eukaryotic viruses.

Quality and adapter trimming on raw paired-end reads was performed using Trimmomatic v0.39 (62). Next, contamination of samples was removed with Bowtie2 v2.3.4 (63) by mapping trimmed reads to a set of contigs present in the negative controls (reagent contamination). The remaining reads were *de novo* assembled into contigs using metaSPAdes v3.13.0 (64). To remove redundancy in the data, contigs were filtered on a length of 1,000 bp and subsequently clustered at 95% nucleotide identity over 80% of the length using Cluster-Genomes (https://bitbucket.org/MAVERICLab/docker-clustergenomes). All contigs were classified by DIAMOND (on the sensitive setting, which can identify sequence similarities of >40%) (65) against the NCBI nr database (downloaded on 27 October 2020) in the sensitive mode for taxonomic annotation. KronaTools (66) was used to parse the DIAMOND output file and find the least common ancestor for each contig (based on the 25 best DIAMOND hits). Contigs annotated as eukaryotic viruses were retrieved using an in-house Python script. Pool magnitudes were obtained by mapping the trimmed and decontaminated reads to these eukaryotic viral contigs with BWA-MEM2 (67, 68). The resulting abundance table was further used for ecological analysis in R using the phyloseq (69), metagenomeSeq (70), vegan (71), and ComplexHeatmap (72) packages.

### Recovery and phylogenetic analysis of (nearly) complete meta-assembled genomes.

To recover full eukaryotic viral genomes in the mosquito pools, viral species were selected based on the level of genome completion after metagenomic *de novo* assembly. If a viral genome was not yet fully complete after assembly, the reads from the mosquito pool were mapped to a selected reference sequence (based on the annotated species using the DIAMOND and Krona tools) with BWA-MEM2 (67, 68). The consensus sequence was subsequently retrieved with samtools and bcftools (73). For phylogenetic analysis, relevant reference complete genome sequences were chosen after BLASTn analysis of the metagenomic assembled genomes (MAGs) and subsequently downloaded from GenBank. Alignment, trimming, model selection, construction, and visualization of phylogenetic trees were done as described above for mosquito COI sequences (see “Mosquito sequence analysis and phylogeny”).

### Data availability.

All mosquito mitochondrial COI sequences obtained in this study have been deposited in GenBank under the accession numbers MW721132 to MW721173. Additionally, the raw sequence reads generated in this study are available in the NCBI Sequence Read Archive (SRA) database under BioProject accession number PRJNA705894. Virus genome sequences retrieved from our samples have been deposited in GenBank under the accession numbers MW699041 to MW699049.
