# Supplementary material for: Establishment of Culex modestus in Belgium and a Glance into the Virome of Belgian Mosquito Species
Source: mSphere. 2021 Apr 21;6(2):e01229-20. doi: 10.1128/mSphere.01229-20 (PMC8546715; doi:10.1128/mSphere.01229-20)
Supplement: TABLE S1 [file msphere.01229-20-st001.pdf]

**Country**	**Locality**	**Accession number (Genbank)**
Spain	Calahorra	MK402890, MK402903
	Viana	MK402912, MK971866, MK971936, MK971952, MK971972
	Hervias	MK402733, MK402793, MK402814, MK402818, MK402875, MK402881, MK402897, MK971950
	Haro	MK402688, MK402692, MK402724, MK402798
	Logrono	MK402689, MK402754, MK402845
Germany	Speyer	MK971796, MK971801, MK971805, MK971807, MK971814, MK971863, MK971869, MK971872, MK971880, MK971911, MK971914, MK971923, MK971931, MK971963, MK971975, MK971989, MK971991, MK972008
	Roememberg	MK971808, MK971828, MK971848, MK971857, MK971861, MK971876, MK971881, MK971902, MK971909, MK971960, MK972005
	Waghausel	MK971883, MK971980
	Ketsch	MK971940
	Trebur	MK971885
	Lingenfeld	MK971824, MK971826, MK971832, MK971858, MK971939, MK971957, MK971983
	Frankfurt	HF562836, HF562837
United Kingdom	Cliffe	JN592733, JN592734, JN592735, MK971798, MK971799, MK971803, MK971809, MK971818, MK971835, MK971845, MK971847, MK971853, MK971860, MK971865, MK971875, MK971878, MK971887, MK971889, MK971893, MK971896, MK971899, MK971905, MK971910, MK971916, MK971920, MK971926, MK971927, MK971935, MK971941, MK971954, MK971966, MK971968, MK971970, MK971982, MK971999, MK972011
	Elmley	MK971995, JN592729, JN592730, KU877022, MK403130, MK403139, MK403162, MK403205, MK403242, MK403257, MK403293, MK403339, MK403462, MK403478, MK971797, MK971819, MK971821, MK971825, MK971827, MK971829, MK971844, MK971846, MK971854, MK971891, MK971904, MK971915, MK971934, MK971942, MK971949, MK971976, MK971994, MK972001, MK972003
	Northward Hill	JN592742, JN592743, JN592744, JN592745
	Kent	MK403327

France	Arles	MK971812, MK971823, MK971833, MK971834, MK971839, MK971852, MK971856, MK971867, MK971870, MK971871, MK971882, MK971903, MK971937, MK971943, MK971947, MK971951, MK971958, MK971967, MK971971, MK971978, MK971981, MK971984, MK971990, MK971996, MK972002
	Camargue	JN592746, JN592747, JN592748
Serbia	Novid sad	MK971874, MK971884, MK971961, MK971998
Portugal	Albufeira	MK971810
	Alcacer	MK971924
Denmark	Greve	KJ401301, KJ401302, KJ401303, KJ401304, KJ401305
	Copenhagen	MK971877, MK971890,
Sweden	Falsterbo	MG844175, MG844176, MG844178
	Simrishamn	MF537266, MG844177
