# Supplementary material for: Establishment of Culex modestus in Belgium and a Glance into the Virome of Belgian Mosquito Species
Source: mSphere. 2021 Apr 21;6(2):e01229-20. doi: 10.1128/mSphere.01229-20 (PMC8546715; doi:10.1128/mSphere.01229-20)
Supplement: TABLE S2 [file msphere.01229-20-st002.pdf]

	Belgium	Denmark	France	Germany	Portugal	Serbia	Spain	Sweden	UK
Hap_1	1								
Hap_2	1								
Hap_3	1								
Hap_4	3								
Hap_5	1								
Hap_6	1								
Hap_7	1								
Hap_8	1	1	3				3		
Hap_9	2								
Hap_10	1								
Hap_11	1								
Hap_12	3		2	2				1	2
Hap_13	1								
Hap_14	2								
Hap_15	1								
Hap_16	1								
Hap_17	1								
Hap_18	2								
Hap_19	1								
Hap_20	3			1					1
Hap_21	1								
Hap_22	1								
Hap_23	1								
Hap_24	2								
Hap_25	1							1	2
Hap_26	1								
Hap_27	2					1			2
Hap_28	1								
Hap_29	1								
Hap_30	1	1						1	
Hap_31	1								
Hap_32	1								
Hap_33	1								
Hap_34		1						1	
Hap_35		1							
Hap_36		1		2					
Hap_37		1							
Hap_38		1							
Hap_39			1						
Hap_40			9						
Hap_41			1						
Hap_42			1						
Hap_43			5						
Hap_44			3						
Hap_45			1						3

Hap_46	1						
Hap_47	1						
Hap_48		9					
Hap_49		9					
Hap_50		2					
Hap_51		3					
Hap_52		1					
Hap_53		4					
Hap_54		1					
Hap_55		1					
Hap_56		1					
Hap_57		1					
Hap_58		2					
Hap_59		1					
Hap_60		1					
Hap_61		1					
Hap_62			1				
Hap_63			1				
Hap_64				1			
Hap_65				1			
Hap_66				1			
Hap_67					2		
Hap_68					5		
Hap_69					1		
Hap_70					8		
Hap_71					1		
Hap_72					1		
Hap_73					1		
Hap_74						1	
Hap_75							1
Hap_76							15
Hap_77							4
Hap_78							1
Hap_79							5
Hap_80							1
Hap_81							1
Hap_82							2
Hap_83							11
Hap_84							6
Hap_85							1
Hap_86							1
Hap_87							1
Hap_88							1
Hap_89							1
Hap_90							2
Hap_91							2

Hap_92	1
Hap_93	2
Hap_94	1
Hap_95	2
Hap_96	1
Hap_97	1
