# Supplementary material for: Establishment of Culex modestus in Belgium and a Glance into the Virome of Belgian Mosquito Species
Source: mSphere. 2021 Apr 21;6(2):e01229-20. doi: 10.1128/mSphere.01229-20 (PMC8546715; doi:10.1128/mSphere.01229-20)
Supplement: TABLE S3 [file msphere.01229-20-st003.pdf]

**Country**	**Locality**	**Haplotypes (n)**
Belgium (2019)	Leuven	Hap1, Hap2, Hap3, Hap4(3), Hap5, Hap7, Hap8, Hap9(2), Hap10, Hap11, Hap12(2), Hap13, Hap14(2), Hap15, Hap16, Hap17, Hap18(2), Hap20(2), Hap22, Hap23, Hap24(2), Hap25, Hap26, Hap27(2), Hap28, Hap29, Hap30, Hap31, Hap32, Hap33,
Belgium (2020)	Leuven	Hap12, Hap19, Hap20, Hap21
Spain	Calahorra	Hap8, Hap68,
	Viana	Hap68(2), Hap70(4),
	Hervias	Hap8, Hap67, Hap70(2), Hap72, Hap73,
	Haro	Hap8, Hap67, Hap69, Hap70(2),
	Logrono	Hap68(2), Hap71,
Germany	Speyer	Hap12(2), Hap36, Hap48(4), Hap49(2), Hap50(2), Hap53, Hap54, Hap56, Hap57, Hap58, Hap60, Hap61,
	Roememberg	Hap20, Hap48, Hap49(3), Hap51(3), Hap53(2), Hap59,
	Waghausel	Hap49, Hap55,
	Ketsch	Hap49,
	Trebur	Hap49,
	Lingenfeld	Hap36, Hap48(2), Hap49, Hap52, Hap53, Hap58,
	Frankfurt	Hap48(2),
United Kingdom	Cliffe	Hap12(2), Hap25, Hap27, Hap45, Hap76(5), Hap77(3), Hap78, Hap79(2), Hap83(7), Hap84(4), Hap90(2), Hap91(2), Hap93(2), Hap94, Hap96, Hap97
	Elmley	Hap20, Hap25, Hap27, Hap45(2), Hap75, Hap76(10), Hap77, Hap79(2), Hap82, Hap83(4), Hap84(2), Hap85, Hap86, Hap87, Hap89, Hap92, Hap95(2)
	Northward Hill	Hap79, Hap80, Hap81, Hap82,
	Kent	Hap88
France	Arles	Hap8(3), Hap12(2), Hap40(8), Hap42, Hap43(5), Hap44(3), Hap45, Hap46, Hap47,
	Camargue	Hap39, Hap40, Hap41,
Serbia	Novid sad	Hap27, Hap64, Hap65, Hap66
Portugal	Albufeira	Hap62
	Alcacer	Hap63
Denmark	Greve	Hap8, Hap30, Hap34, Hap35, Hap36,
	Copenhagen	Hap37, Hap38,
Sweden	Falsterbo	Hap12, Hap34, Hap74,
	Simrishamn	Hap25, Hap30,
