# Supplementary material for: Establishment of Culex modestus in Belgium and a Glance into the Virome of Belgian Mosquito Species
Source: mSphere. 2021 Apr 21;6(2):e01229-20. doi: 10.1128/mSphere.01229-20 (PMC8546715; doi:10.1128/mSphere.01229-20)
Supplement: TABLE S4 [file msphere.01229-20-st004.pdf]

**Virus**	**Closest match (Blastx)**	**Accession number**	**% Per. Identity (aa)**	**Genome size (bp)**
Culex totivirus Leu1	Culex inatomii totivirus (capsid protein)	LC514398.1	98.3	6241
Culex totivirus Leu2	Culex inatomii totivirus (capsid protein)	LC514398.1	98.3	6273
Culex totivirus Leu3	Culex inatomii totivirus (capsid protein)	LC514398.1	98.2	6200
Alphamesonivirus Leu 4	Alphamesonivirus 1 (pp1ab polyprotein)	MH520101.1	99.7	20153
Iflavirus Leu5	Culex iflavi-like virus 4 (polyprotein)	MT096522.1	98.3	9634
Iflavirus Leu6	Yongsan iflavirus 1 (polyprotein)	NC_040587.1	97.1	8977
Negevirus Leu7	Yongsan negev-like virus 1 (RdRp)	MH703054.1	94.6	8388
Negevirus Leu8	Yongsan negev-like virus 1 (RdRp)	MH703054.1	95.6	8312
Rhabdovirus Leu9	Riverside virus 1 (large protein)	KU248086.1	98.2	11659
