# Supplementary material for: Establishment of Culex modestus in Belgium and a Glance into the Virome of Belgian Mosquito Species
Source: mSphere. 2021 Apr 21;6(2):e01229-20. doi: 10.1128/mSphere.01229-20 (PMC8546715; doi:10.1128/mSphere.01229-20)
Supplement: TABLE S5 [file msphere.01229-20-st005.pdf]

**Viral species annotation**	**Contig name**	**Blastx % identity**
Alphamesonivirus 1	NODE_1_length_15647_cov_3928.343417_Pool1-Culexurban	99.7
	NODE_8_length_4621_cov_5204.996479_Pool1-Culexurban	99.2
Atrato Partiti-like virus 3	NODE_132_length_1289_cov_35.661716_Pool4-Culexperiurban1	80.1
Atrato Retro-like virus	NODE_14_length_3433_cov_40.791120_Pool2-Culexurban	42.6
	NODE_438_length_1086_cov_6.878097_Pool5-Culexperiurban2	39.3
	NODE_884_length_1159_cov_19.977819_Pool2-Culexurban	58.6
Atrato Sobemo-like virus 1	NODE_36_length_2400_cov_12.924666_Pool8-Aedes2	90
Beaumont virus	NODE_71_length_1624_cov_30.435036_Pool3-anopheles_culiseta	49.4
Beihai partiti-like virus 2	NODE_147_length_1439_cov_82.527900_Pool5-Culexperiurban2	31.3
	NODE_199_length_1347_cov_206.896063_Pool5-Culexperiurban2	35.7
Botrytis cinerea hypovirus 1	NODE_28_length_2705_cov_4.025875_Pool1-Culexurban	97.9
	NODE_44_length_2109_cov_6.711122_Pool1-Culexurban	98.3
Broome chryso-like virus 1	NODE_18_length_3356_cov_29.729491_Pool1-Culexurban	91.9
	NODE_69_length_1832_cov_30.791453_Pool5-Culexperiurban2	68.8
Chaq virus-like 1	NODE_104_length_1373_cov_3480.986883_Pool3-anopheles_culiseta	31.2
Chibugado virus	NODE_130_length_1262_cov_23.807595_Pool3-anopheles_culiseta	50.6
	NODE_173_length_1164_cov_11.518859_Pool3-anopheles_culiseta	53.7
	NODE_380_length_1067_cov_4.237374_Pool8-Aedes2	51
	NODE_75_length_1572_cov_14.608027_Pool3-anopheles_culiseta	25.8
Culex Iflavi-like virus 4	NODE_1_length_8540_cov_675.062862_Pool2-Culexurban	98.1
Culex inatomii totivirus	NODE_3_length_6273_cov_354.762912_Pool5-Culexperiurban2	98.3
Culex orthomyxo-like virus	NODE_46_length_2056_cov_40.703891_Pool5-Culexperiurban2	91.8

dsRNA virus environmental sample	NODE_28_length_2378_cov_13.866580_Pool3-anopheles_culiseta	59.6
	NODE_35_length_2290_cov_16.011749_Pool3-anopheles_culiseta	66.4
	NODE_38_length_2372_cov_14.497168_Pool8-Aedes2	48.5
	NODE_61_length_1969_cov_10.223573_Pool8-Aedes2	44.3
	NODE_80_length_1753_cov_7.751790_Pool8-Aedes2	43
Embera virus	NODE_7_length_4334_cov_96.735494_Pool8-Aedes2	56.4
Fitzroy Crossing toti-like virus 2	NODE_12_length_3706_cov_123.676495_Pool5-Culexperiurban2	79.2
Flen bunya-like virus	NODE_263_length_1056_cov_6.667007_Pool7-Aecinereus1	34.9
Hubei chryso-like virus 1	NODE_16_length_3037_cov_985.481757_Pool3-anopheles_culiseta	44.8
	NODE_17_length_3010_cov_1343.402659_Pool3-anopheles_culiseta	42.2
	NODE_22_length_2995_cov_26.976696_Pool1-Culexurban	80.5
	NODE_258_length_1336_cov_6.681493_Pool1-Culexurban	75.8
	NODE_25_length_3024_cov_26.126909_Pool8-Aedes2	40
	NODE_489_length_1110_cov_7.539206_Pool1-Culexurban	64.25
	NODE_72_length_1843_cov_14.233296_Pool1-Culexurban	86.6
Hubei partiti-like virus 22	NODE_56_length_1806_cov_697.011567_Pool3-anopheles_culiseta	88
Hubei partiti-like virus 35	NODE_236_length_1054_cov_41.616172_Pool3-anopheles_culiseta	65.1
Hubei toti-like virus 10	NODE_90_length_1400_cov_17.414966_Pool7-Aecinereus1	58.7
Ista virus	NODE_133_length_1288_cov_18.982659_Pool4-Culexperiurban1	81.6
	NODE_151_length_1427_cov_24.004444_Pool5-Culexperiurban2	86.3
	NODE_166_length_1218_cov_18.815074_Pool4-Culexperiurban1	68.8
	NODE_267_length_1079_cov_24.114770_Pool4-Culexperiurban1	77.3
	NODE_342_length_1165_cov_11.166360_Pool5-Culexperiurban2	84.5
Partitivirus-like 2	NODE_69_length_1640_cov_148.561740_Pool3-anopheles_culiseta	70.2

Riverside virus 1	NODE_23_length_3135_cov_20.394048_Pool8-Aedes2	99.1
	NODE_367_length_1079_cov_12.566866_Pool8-Aedes2	100
	NODE_58_length_2004_cov_33.143228_Pool8-Aedes2	100
	NODE_8_length_4204_cov_88.106373_Pool8-Aedes2	99.8
Salado virus	NODE_15_length_3182_cov_1163.568760_Pool3-anopheles_culiseta	41.8
	NODE_22_length_3162_cov_26.363047_Pool8-Aedes2	36.1
Shuangao insect-associated chrysovirus	NODE_14_length_3528_cov_179.931614_Pool8-Aedes2	59.7
	NODE_66_length_1933_cov_17.809806_Pool8-Aedes2	47.9
Sonnbo virus	NODE_79_length_1746_cov_420.109047_Pool5-Culexperiurban2	97.8
Whidbey virus	NODE_54_length_1655_cov_22.948669_Pool7-Aecinereus1	85.8
Wuhan insect virus 23	NODE_100_length_1389_cov_65.072409_Pool3-anopheles_culiseta	37.3
Wuhan Mosquito Virus 6	NODE_1133_length_1099_cov_22.933464_Pool2-Culexurban	97.35
	NODE_294_length_1212_cov_12.079295_Pool5-Culexperiurban2	28.4
	NODE_649_length_1249_cov_15.440273_Pool2-Culexurban	95.6
Yongsan iflavirus 1	NODE_16_length_3251_cov_536.527410_Pool2-Culexurban	98.9
	NODE_2_length_5940_cov_4261.994713_Pool2-Culexurban	96.9
	NODE_32_length_2236_cov_24.789717_Pool5-Culexperiurban2	80.5
Yongsan negev-like virus 1	NODE_1_length_10958_cov_1397.451153_Pool5-Culexperiurban2	95.1
