# Supplementary figures and images for: Establishment of Culex modestus in Belgium and a Glance into the Virome of Belgian Mosquito Species
Source: mSphere. 2021 Apr 21;6(2):e01229-20. doi: 10.1128/mSphere.01229-20 (PMC8546715; doi:10.1128/mSphere.01229-20)

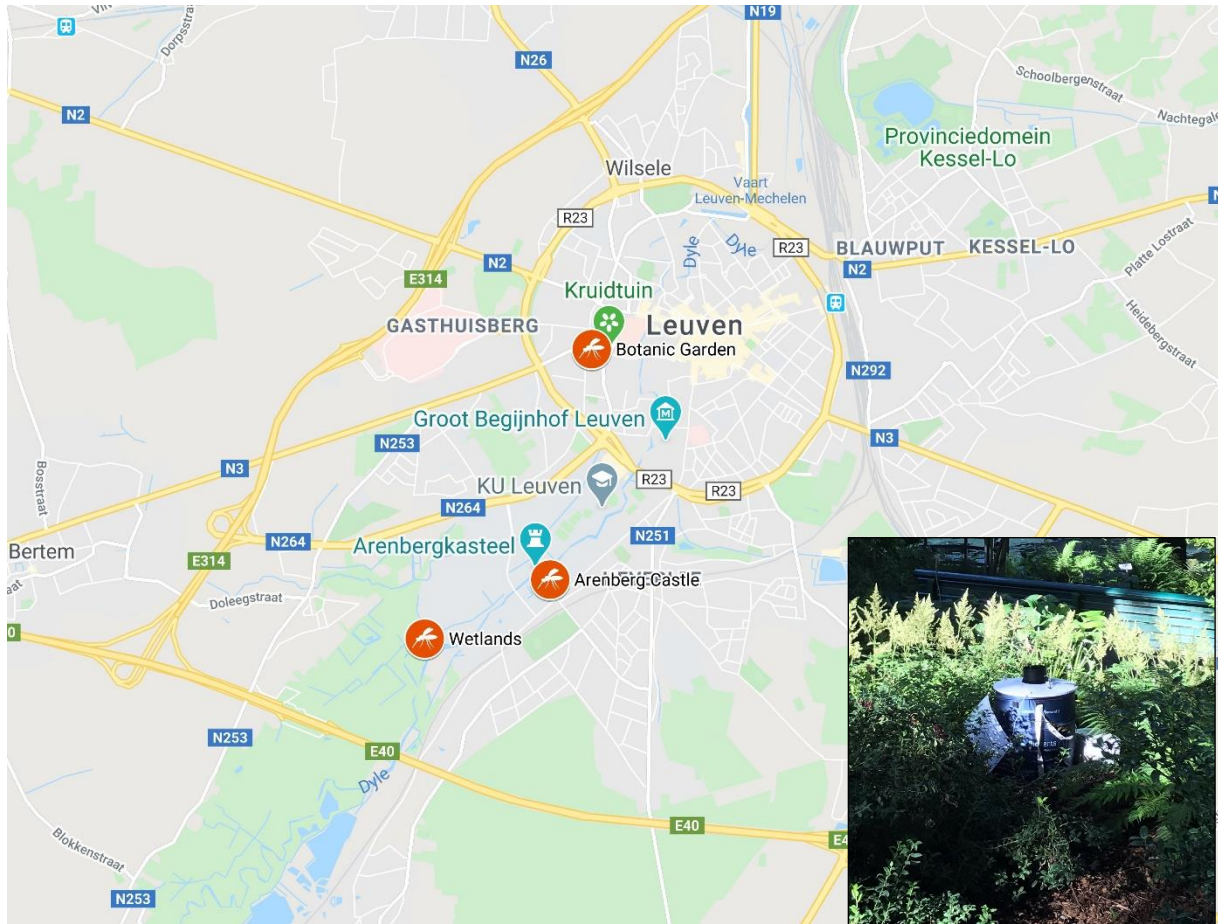

Supplement: FIG S1 [file msphere.01229-20-sf001.pdf]
